# Comment on “nontuberculous mycobacterial infection after lung transplantation: a report of four cases”

**DOI:** 10.1186/s40792-019-0676-8

**Published:** 2019-07-23

**Authors:** Masoud Keikha, Kiarash Ghazvini

**Affiliations:** 1 Antimicrobial Resistance Research Center, Mashhad University of Medical Sciences, Mashhad, Iran; 2 Department of Microbiology and Virology, Faculty of Medicine, Mashhad University of Medical Sciences, Mashhad, Iran

**Keywords:** Nontuberculous mycobacteria (NTM), Infection, Lung, Transplantation, Treatment, Molecular identification

## Abstract

Nontuberculous mycobacteria can lead to pulmonary infection in lung transplantation cases, which is associated with high mortality rates. We hereby thank Dr. Ose et al. very much for their report on “Nontuberculous mycobacterial infection after lung transplantation: A report of four cases”; however, we have confronted with several questions to have a new review of these cases.

Dear Editor;

Dr. Ose et al. recently published their report on “Nontuberculous mycobacterial infection after lung transplantation: A report of four cases” [1]. Nontuberculous mycobacteria (NTM) are saprophytes, which are free-living organisms in the surrounding environment such as water, soil, milk, dust, decaying vegetables, and animals [2]. NTM were considered as non-pathogenic mycobacteria until the first report by Prissick and Masson in the 1950s about cervical lymphadenitis caused by chromogenic mycobacteria [3]. There have been numerous evidences in recent years for the isolation of NTM from hospital environment sources, and it is suggested that NTM spp. are transmitted to the hospitalized patients throughout contaminated ventilators, aerosols, catheters, and medical equipment, causing NTM infections in susceptible individuals, including AIDS (acquired immunodeficiency syndrome), cancer, transplantation, or in the immune-compromised patients [4].

According to the American Thoracic Society (ATS) recommendations, NTM infections should be identified to the species level, because of clinical significance, patient management, appropriate treatment, and epidemiological goals; based on the ATS criteria, NTM pulmonary infection is diagnosed according to a single isolation of NTM spp. from bronchoalveolar lavage (BAL) or pulmonary biopsy, the isolation of NTM spp. in at least two separate sputum specimens, or clinical symptoms with radiographic findings [5, 6]. According to review of literatures, treatment options for NTM infections are different from *Mycobacterium tuberculosis* (*Mtb*) infection. In addition, NTM spp. are subdivided into two categories of rapid-growing mycobacteria (RGM) and slow growing mycobacteria (SGM); unfortunately, RGM are resistant to the therapeutic option of SGM infection. Therefore, identification of NTM to the species level plays a key role in appropriate treatment of NTM infection [7]. However, the emergence of drug-resistant species, particularly *M. fortuitum*, *M. chelonae*, and *M. abscessus*, has serious challenges and is associated with a high rate of mortality [8].

Conventional identification of NTM species, considering the factors such as growth rate, pigment production, growth on MacConkey agar, iron uptake, urease production, tween 80 hydrolysis, nitrate reduction, niacin production, catalase, tellurite reduction, aryl sulfatase (3 days), sensitivity to antimicrobial agents, or HPLC (high-performance liquid chromatography) analysis of mycolic acid, is expensive and labor-intensive, needs trained technicians, and takes several weeks for the assessments. However, molecular methods including sequencing, line probe assay, PCR-RFLP (polymerase chain reaction-restriction fragment length polymorphism), or LAMP (loop-mediated isothermal amplification) using housekeeping genes of 16S rRNA, *hsp65*, *rpoB*, *secA1*, *tuf*, *gyrB*, or *recA* are reliable methods for rapid, inexpensive, and accurate identification of NTM species, particularly in emergence of disseminated infections [9].

There are several case reports of NTM infections in lung transplantation issues; Valentine et al. have shown that *Mycobacterium abscessus*, *Mycobacterium avium* complex, *Mycobacterium gordonae*, *Mycobacterium chelonae*, and *Mycobacterium fortuitum* are common NTM species, which are isolated from pulmonary infection of lung transplantation cases besides multiple NTM infections [10]. In addition, it is estimated that 0.46–9% of lung transplantation were fallen to NTM infection, in relation with the transplant rejection or high mortality rates (even 44% mortality) [6, 11, 12]. We should thank Dr. Ose et al. very much for this article, and it is presumed that developing these studies can be useful for the appropriate management of NTM infection of transplantation cases. However, we hereby request the authors to pay attention to the following issues:

1. Please describe the mycobacterial isolation method, which was not stated in the report.

In the diagnosis of NTM infection in a lung transplant patient, it can be noticed that the infection can occur by contamination or transient colonization in the conventional method by bronchoalveolar lavage or biopsy and sputum-positive culture. This case did not show a clinical symptom and completely recovered with no chemotherapy, which could be considered as transient colonization

2. Please explain how the Mycobacterium spp. were finally identified to the species level that was not declared in this report.
3. What is the basis of the antibiotic regimen selection, whereas there is no information about performing an antibiotic susceptibility test in this report?
4. Please describe the previous infection of the lung transplantation cases and also give more details about the clinical symptoms of the cases.
5. Please tell us about the role of this bacterium in the lung transplantation and also about the lack of relevance of the lung transplantation infections with other bacteria.

## Data Availability

There were no available repositories.

## Abbreviations

AIDS: Acquired immunodeficiency syndrome
ATS: American Thoracic Society
HPLC: High-performance liquid chromatography
LAMP: Loop-mediated isothermal amplification
*Mtb*: *Mycobacterium tuberculosis*
Mycobacterium spp.: Mycobacterial species
NTM: Non-tuberculosis mycobacteria
PCR-RFLP: Polymerase chain reaction-restriction length polymorphism
RFLP: Restriction fragment length polymorphism
RGM: Rapid-growing mycobacteria
SGM: Slow-growing mycobacteria
